# Principles of Chemistry

**Published:** 1853-03

**Authors:** 


### REVIEWS.

---

Art. VI.—*Principles of Chemistry.* By B Silliman, Jr., A. M., M. D., Professor of Chemistry and Toxicology, in the University of Louisville. With 423 Illustrations. Philadelphia: Peck & Bliss.

We learn from the title-page that this excellent manual has reached the *twenty-fifth* edition. We confess ourselves both surprised and gratified by this intelligence. When it is remembered that the first edition was published only six years ago, that with the judicious few it has had to compete with the standard works of Graham and Fownes, while the tide of popular preference has always set strongly in favor of the cheap and trashy compilation of Comstock, &c. This marked success must be regarded as evidence of the intrinsic value of the book itself, as it also indicates that the public begin to feel the necessity of having more scholarly school-books. In regard to the science of Chemistry, this work of Professor Silliman satisfies the growing demand of the public, which indeed it has materially aided in creating. The author's early education in the laboratory, his long and successful experience as a teacher and lecturer, and his familiarity with the applications of chemistry to the arts, and with its kindred sciences have eminently qualified him to write a text-book of standard excellence. The judicious selection of topics, and their arrangement so that the student may pass easily from one to another, show that the author is not a mere compiler of other men's researches, but an original investigator, and practically acquainted with the subject whereof he writes. The young student, who distracted by complications of glass tubes and wolfian bottles, sees nothing in a brilliant experiment but a flash, fizzle, and smoke, may clear up the mystery if he will turn to the clear and precise explanations of Professor Silliman. At least, he will not have to contend with any difficulties created by the deficient ability of the author, and imposed on those which are inherent in the science itself.

Although the present edition has been entirely re-written so as to incorporate the numerous discoveries of the last five years, still the size of the volume has not been much increased. The condensation of the subject is of that admirable kind which omits no important fact or principle, while irrevelant or merely supplimentary matter is rigorously excluded. In compliance with the custom of English and American writers, which, indeed, the defective education of nearly all chemical students has rendered necessary, the first 149 pages of this volume are devoted to those elementary principles of Physics, in particular, of Heat and the Protean forms of Electricity, which are most important to be known previous to the study of Chemistry. The next 18 pages contain the laws of chemical combination and the nomenclature, and the classification of the elements, 62 in number; the history of these and their important combinations occupies 208 pages, and the remainder is filled by an original treatise on the most intricate part of the science —Organic Chemistry. This has been prepared by one of the most accomplished chemists of our country, T. S. Hunt, who was formerly the pupil and associate of Prof. Silliman.

In conclusion we must award their merited praise to the publishers for the superior quality of the printing and binding of this work. The number of illustrations has been more than doubled. Many of them were drawn for this edition from the author's apparatus, and these, as it is hardly necessary to say, are models of philosophical neatness and elegance.

The author appropriately dedicates his work to his distinguished father, Benjamin Silliman, Senior, for fifty years Professor of Chemistry in Yale College. It is a just tribute, as well of filial affection, and of that respect and veneration which every lover of science feels for him, who has done more than any other Amercan for the interests of American science, whose eloquent lectures and writings having made science popular and acceptable with all classes of his countrymen. Long may he live, surrounded by "troops of friends," to enjoy in his serene old age the honors, reward and affection, which follow a long life devoted to noble studies, and gratefully yielded to the "Father of American Science."

